# PECAM-1 engagement counteracts ICAM-1-induced signaling in brain vascular endothelial cells^2^

**DOI:** 10.1111/j.1471-4159.2007.04782.x

**Published:** 2007-10

**Authors:** Jean-Pierre Couty, Christine Rampon, Mathilde Leveque, Marie-Pierre Laran-Chich, Sandrine Bourdoulous, John Greenwood, Pierre-Olivier Couraud

**Affiliations:** * Institut Cochin, Université Paris DescartesCNRS (UMR 8104), Paris, France; † INSERMParis, France; ‡ Division of Cell Biology, Institute of Ophthalmology, University College LondonLondon, UK

**Keywords:** adhesion molecules, endothelial cells, ICAM-1, PECAM-1, signaling pathways

## Abstract

Interactions between leukocytes and vascular endothelial cells are mediated by a complex set of membrane adhesion molecules which transduce bi-directional signals in both cell types. Endothelium of the cerebral blood vessels, which constitute the blood–brain barrier, strictly controls adhesion and trafficking of leukocytes into the brain. Investigating signaling pathways triggered by the engagement of adhesion molecules expressed on brain endothelial cells, we previously documented the role of ICAM-1 in activation of the tyrosine phosphorylation of several actin-binding proteins and subsequent rearrangements of the actin cytoskeleton. In the present study, we show that, whereas PECAM-1 is known to control positively the trans-endothelial migration of leukocytes via homophilic interactions between leukocytes and endothelial cells, PECAM-1 engagement on brain endothelial surface unexpectedly counteracts the ICAM-1-induced tyrosine phosphorylation of cortactin and rearrangements of the actin cytoskeleton. We present evidence that the PECAM-1-associated tyrosine phosphatase SHP-2 is required for ICAM-1 signaling, suggesting that its activity might crucially contribute to the regulation of ICAM-1 signaling by PECAM-1. Our findings reveal a novel activity for PECAM-1 which, by counteracting ICAM-1-induced activation, could directly contribute to limit activation and maintain integrity of brain vascular endothelium.

Leukocyte extravasation into tissues involves a complex set of adhesion molecules at the surface of leukocytes and vascular endothelial cells. Tethering or rolling of leukocytes is followed by their firm adhesion to endothelium, their locomotion (Schenkel *et al.* 2004) at the endothelial surface, which itself precedes diapedesis (Butcher 1991). In the central nervous system, brain endothelial cells are joined by continuous tight junctions, constituting the blood–brain barrier (BBB), which strictly limits leukocyte infiltration, as well as drug access to the cerebral compartment. Nevertheless, in pathological situations, such as multiple sclerosis, viral or bacterial infections, numerous activated lymphocytes, monocytes or neutrophils can cross the BBB (Carson *et al.* 2006; Engelhardt 2006).

On the endothelial apical surface, ICAM-1 is a key player in firm adhesion and locomotion steps. In addition, PECAM-1, which is expressed in endothelial cells, monocytes, neutrophils and specific T lymphocyte subsets, is directly involved in diapedesis via homophilic interactions between migrating leukocytes, particularly monocytes/neutrophils and endothelial intercellular junctions (Muller *et al.* 1993). Paradoxically, however, gene deficiency for PECAM-1 was recently found to increase the number of activated leukocytes crossing the BBB, suggesting that PECAM-1 might play a more complex role in leukocyte extravasation than previously recognized (Graesser *et al.* 2002).

These adhesion molecules have been well documented as signal transducers in leukocytes and endothelial cells, in as much as leukocyte adhesion to endothelial cells as well as antibody cross-linking were shown to activate multiple signaling pathways in both cell types. Using brain endothelial cell lines, we previously provided evidence that ICAM-1 antibody cross-linking led to an increase in intracellular Ca^2+^ concentration, protein kinase C activation, phosphorylation of cortactin and other actin-binding proteins by the Src tyrosine kinase, activation of RhoA GTPase, and subsequent rearrangements of the actin cytoskeleton (Durieu-Trautmann *et al.* 1994; Greenwood *et al.* 2002; Carman and Springer 2004; Shaw *et al.* 2004; Yang *et al.* 2005; Millan *et al.* 2006). Besides, PECAM-1 has been abundantly documented as a signaling receptor which can transduce either inhibitory or stimulatory signals with cell-specificity, such as inhibition of the antigen receptor signaling in T lymphocytes or stimulation of the intracellular calcium level in endothelial cells (Newman *et al.* 2001; Newman and Newman 2003).

However, no evidence to our knowledge has emerged on how the two activated signaling pathways coupled to ICAM-1 and PECAM-1 are integrated by endothelial cells and to what extent they might contribute in a sequential and coordinated manner to the endothelial response to leukocyte adhesion. In the present study, we addressed the question of a putative cross-talk between these two signaling pathways by sequential antibody cross-linking of ICAM-1 and PECAM-1 at the surface of endothelial cells: this experimental approach has been shown by us and others to mimic leukocyte interaction with endothelial cells and to allow the biochemical analysis of endothelial response to leukocyte adhesion. The rat brain endothelial cell line RBE4 was used here as a robust model of brain microvascular endothelium (Schweitzer *et al.* 1997; Hoffmann *et al.* 2001); We report in the present study that PECAM-1 engagement unexpectedly down-regulated ICAM-1-induced tyrosine phosphorylation of cortactin and rearrangements of the actin cytoskeleton. The functional relevance of this finding is discussed in terms of regulation of BBB integrity in inflammatory situations.

## Materials and methods

### Abs and reagents

Mouse mAb to rat ICAM-1 (clone 1A29), Major Histocompatibility Complex (MHC) class II (OX6) and Transferrin receptor (OX26) were purchased from Serotec (Wiesbaden, Germany). Anti-PECAM-1 mAb 4E8 and anti-ICAM-1 mAb 3H8 were kindly provided by Dr Hickey (Darmouth Medical School, Hanover, NH, USA). M20 polyclonal Abs to rat PECAM-1, anti-SHP2 and anti-RhoA mAb were purchased from Santa Cruz Biotechnology (Santa Cruz, CA, USA). Rabbit anti-mouse (RAM) Abs were from DaKo France (Trappes, France). Anti-phosphotyrosine and -cortactin mAbs were purchased from Upstate Biotechnology/Millipore (Billerica, MA, USA). Calpeptin was purchased from Calbiochem (La Jolla, CA, USA). Tetramethylrhodamine isothiocyanate-conjugated phalloidin was purchased from Sigma–Aldrich (St Louis, MO, USA).

### Endothelial cell lines

The rat brain microvascular endothelial (RBE4) cell line was produced by us and extensively characterized (Roux *et al.* 1994; Etienne-Manneville *et al.* 2000). RBE4 cells were grown as previously described (Etienne-Manneville *et al.* 2000). The human bone marrow microvascular endothelial (HBMEC) cell line (Schweitzer *et al.* 1997) was kindly provided by Dr B. Weksler (Weill Medical College of Cornell University, New York). HBMECs were cultured as previously described (Hoffmann *et al.* 2001). Both cell lines maintain in culture a fully differentiated endothelial phenotype.

Generation of HBMEC stable transfectants: HBMECs were transfected with either the pcDNA3.1-SHP-2 (human wild type) or pcDNA3.1-DnSHP-2 (Dominant negative) or “empty” plasmid (“mock”) kindly provided by Dr C. Nahmias (Rivard *et al.* 1995) using the nucleofector system developed by Amaxa Inc (Gaithersburg, MD, USA). Briefly, for optimal transfection of HBMECs, 10^6^ cells were suspended in 100 μL of solution V provided by Amaxa in the presence of 1.5 μg DNA, and subjected to electroporation using the program U15 of the nucleofector system. Cells were replated in complete medium containing 10% fetal calf serum. The day after 400 μg/mL active G418 (Invitrogen, Cergy Pontoise, France) were added to the cells. In order to obtain clonal transfectants, selected neomycin plates were split to obtain isolated colonies. The clones and pools (containing multiple clones) were screened for SHP-2 expression by western blotting with specific anti-SHP2 antibodies. One pool and several clones expressing the dominant negative form of SHP-2 (HBMEC/Dn-SHP-2) or wild type SHP-2 (HBMEC/WT-SHP-2) and one pool transfected with empty plasmid (HBMEC/mock) were chosen for further studies. Stable transfectants were maintained in G418 and passaged before attaining confluence.

### Ab cross-linking of ICAM-1 and PECAM-1

Confluent endothelial cells were pre-incubated with serum- and growth factor-free culture medium containing 100 U/mL IFN-γ for 48 h, before Ab cross-linking of ICAM-1 and/or PECAM-1. This treatment has been shown to increase significantly the surface expression of ICAM-1 in RBE4 cells and was thus included in the ICAM-1 cross-linking procedure, as described (Etienne *et al.* 1998; Etienne-Manneville *et al.* 2000; Greenwood *et al.* 2002). In addition, it was also previously reported to induce MHC II surface expression in the same cells (Etienne *et al.* 1999).

Ab cross-linking was performed in four sequential steps at 37°C, under a 5% CO_2_ atmosphere, as summarized in Table 1: (i) cells were incubated for 15-min with serum-free medium alone (–) or with anti-ICAM-1 antibody (5 or 10 μg/mL); (ii) after two washes with serum-free medium alone to remove unbound antibodies, cells were incubated for 10-min with medium alone (–) or with rabbit anti-mouse (RAM 10 μg/mL) antibodies; (iii) cells were next washed twice and incubated for 15-min with serum-free medium alone (–), with anti-PECAM-1 antibody (10 μg/mL) or with an isotype-matched anti-MHC II antibody (10 μg/mL); (iv) finally, after two washes, cells were incubated for 10-min with medium alone (–) or with RAM antibodies (10 μg/mL).

**Table 1:** Experimental procedure for sequential cross-linking of ICAM-1 and/or PECAM-1 at the surface of cultured endothelial cells

Steps	1 (15 min)	2 (10 min)	3 (15 min)	4 (10 min)
Conditions				
BASAL	–	RAM	–	RAM
ICAM-1	α-ICAM-1	RAM	–	RAM
PECAM-1	–	–	α-PECAM-1	RAM
ICAM-1 + PECAM-1 (I + P)	α-ICAM-1	RAM	α-PECAM-1	RAM
PECAM-1 + ICAM-1 (P + I)	α-PECAM-1	RAM	α-ICAM-1	RAM
ICAM-1 + MHC-II	α-ICAM-1	RAM	α-MHC-II	RAM

The anti-MHC II antibody used here as an isotype-matched control antibody was previously shown not only to bind to IFN-γ-activated RBE4 cells, but also to trigger signaling pathways in RBE4 cells distinct from those activated by ICAM-1 cross-linking (Durieu-Trautmann *et al.* 1994; Etienne *et al.* 1999; Greenwood *et al.* 2002).

In order to inhibit SHP-2 phosphatase, endothelial cells (RBE-4 or HBMECs) were treated with 150 μg/mL of calpeptin (Calbiochem, La Jolla, CA, USA) for 30 min prior to ICAM-1 cross-linking.

### Immunofluorescence analysis

Cells grown to confluence on Permanox coverslips (Costar, Corning, NY, USA), were starved in serum-and growth factor-free medium for 24 h, and then treated as described above for antibody cross-linking. F-actin staining was performed as previously described (Etienne-Manneville *et al.* 2000) and fluorescence images were collected using a scanning confocal microscope (MCR. 1000; Bio-Rad, Hercules, CA, USA). At least three independent experiments were performed in triplicates.

### Immunoprecipitations and western blotting analysis

Following treatments, cells were washed with ice-cold phosphate-buffered saline and lysed for 30 min at 4°C for – cortactin and ICAM-1 experiments: in Nonidet P-40 buffer containing 10 mmol/L Tris–HCl, pH 7.5, 140 mmol/L NaCl, 1% NP-40, 1 mmol/L phenylmethylsulfonyl fluoride, 1 mmol/L orthovanadate, 50 units/mL aprotinin, 1 mmol/L EDTA, 2 μg/mL pepstatin, 2 μg/mL leupeptin.

PECAM-1 experiments: cells were lysed in Nonidet P-40 buffer containing 50 mmol/L Tris–HCl, pH 7.5, 250 mmol/L NaCl, 0.5% NP-40, 5 mmol/L EDTA, 1 mmol/L CaCl_2_, 1 mmol/L MgCl_2_, supplemented with the same protease inhibitors used in cortactin lysis buffer.

Nuclei were discarded following centrifugation at 13 000 *g* for 10 min at 4°C. Cleared lysates were incubated for 3 h at 4°C with specific Abs, then for 2 h with agarose beads which were pre-incubated for 2 h with an excess of control mouse immunoglobulins. Immunoprecipitates were collected by centrifugation at 1200 *g* and processed for western blotting analysis as previously described (Etienne-Manneville *et al.* 2000). At least three independent experiments were performed in triplicates.

### RhoA activity assay

Cells were lysed in 50 mmol/L Tris pH 7.5, 150 mmol/L NaCl, 10% glycerol, 1% NP-40, 0.1% Triton X-100, 5 mmol/L MgCl_2_, 0.1 mmol/L phenylmethylsulfonyl fluoride, 10 μg/mL leupeptine, 10 μg/mL aprotinin. Cleared lysates were incubated with bacterially produced GST-RBD (Rhotekin) bound to glutathione–agarose beads for 45 min at 4°C, as described (Reid *et al.* 1996; Sander and Collard 1999). The beads were washed three times with lysis buffer, and then bound proteins were analyzed by SDS–PAGE and western blotting as described (Etienne *et al.* 1998). At least three independent experiments were performed in triplicates.

## Results

### PECAM-1 cross-linking inhibits ICAM-1-induced actin cytoskeleton rearrangements

In order to assess the existence of a putative cross-talk between the signaling pathways coupled to ICAM-1 and PECAM-1 in brain endothelial cells, we developed a procedure of sequential antibody cross-linking of ICAM-1 and PECAM-1 (see Materials and methods) to mimic the supposed sequence of events supporting leukocyte extravasation.

**Fig. 1:**
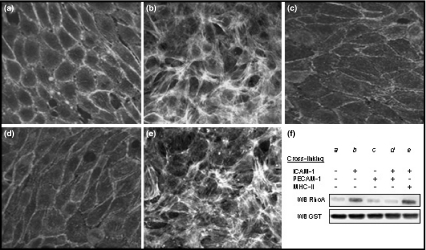
PECAM-1 cross-linking reverses ICAM-1-induced actin cytoskeleton rearrangements. Sequential cross-linking of ICAM-1 and/or PECAM-1 was performed on IFN-γ-treated RBE4 cells as described in Material and methods. Panels (a–e) Cells were fixed and actin was stained by using 0.1 μg/mL of tetramethylrhodamine isothiocyanate-labeled phalloidin. Panel (a) basal condition. Panel (b) ICAM-1 cross-linking. Panel (c) PECAM-1 cross-linking. Panel (d) Sequential ICAM-1 and then PECAM-1 cross-linking. Panel (e) As a control, sequential ICAM-1 and then MHC-II cross-linking. Panel (f) GST-Rhotekin pull-down. Proteins were eluted with SDS-sample buffer, and resolved on a 12% SDS–PAGE followed by immunoblotting with anti-RhoA specific or anti-GST antibody. Lane a: basal condition; lane b: ICAM-1 cross-linking; lane c: PECAM-1 cross-linking; lane d: sequential ICAM-1 and then PECAM-1 cross-linking; lane e: sequential ICAM-1 and then MHC-II cross-linking.

We previously documented that ICAM-1 cross-linking at the surface of the rat brain endothelial RBE4 cells lead to actin cytoskeleton rearrangements through activation of RhoA GTPase (Etienne *et al.* 1998; Adamson *et al.* 1999; Etienne-Manneville *et al.* 2000; Yang *et al.* 2006). Here, we confirmed that, whereas polymerized actin was mainly cortical in basal condition (incubation with Rabbit anti-Mouse antibodies) (Fig. 1a), ICAM-1 cross-linking induced the formation of numerous actin stress fibers across RBE4 cells (Fig. 1b); this response was maintained over the entire incubation period (50 min). By contrast, no stress fiber formation was observed following PECAM-1 cross-linking (Fig. 1c). Unexpectedly, we did not observe any stress fiber formation following sequential antibody cross-linking of ICAM-1 and PECAM-1 (Fig. 1d). As control, cross-linking of ICAM-1 followed by incubation with buffer alone or with the isotype-matched anti-MHC-II antibody (Fig. 1e) or anti-transferrin antibody (not shown) or again with the same anti-ICAM-1 antibody (not shown) did not affect the ICAM-1-induced formation of stress fibers. Similar results were observed using the human microvascular endothelial cell line HBMEC of peripheral origin (not shown). These results indicate that PECAM-1 engagement drastically blocked the ICAM-1-induced cortical actin polymerization in RBE4 cells and human endothelial cells.

Because actin stress fiber formation is known as a RhoGTPase-mediated process, we evaluated this activity in RBE4 cell extracts in the same conditions of ICAM-1 and/or PECAM-1 antibody cross-linking. For that purpose, we used a validated pull-down assay based on the specific interaction between the activated GTP-bound RhoA (compared to the non-activated GDP-bound RhoA) and the RhoA-binding domain (RBD) of the RhoA effector Rhotekin fused to glutathione-S-transferase (GST) (Reid *et al.* 1996; Sander and Collard 1999). Following ICAM-1 cross-linking or control treatment, cell extracts containing equal amounts of RhoA protein (as confirmed by western blotting: not shown) were incubated with the the Rhotekin-GST fusion protein: a large amount of retained (i.e. activated) RhoA was detected following ICAM-1 cross-linking (Fig. 1f, lane b), compared to basal conditions (Fig. 1f, lane a), confirming our previous finding that ICAM-1 cross-linking induces a strong activation of RhoA in RBE4 cells. PECAM-1 cross-linking *per se* did not activate RhoA (Fig. 1f, lane c), but completely inhibited ICAM-1-induced RhoA activation (Fig. 1f, lane d). As a control, the irrelevant isotype-matched anti-MHC-II antibody had no effect on ICAM-1 response (Fig. 1f, lane e). These results provide further evidence that PECAM-1 cross-linking affects the ICAM-1-induced cortical actin polymerization by interfering with RhoA activation.

### PECAM-1 cross-linking inhibits ICAM-1-induced cortactin phosphorylation

Together with actin polymerization, we previously documented that ICAM-1 cross-linking strongly stimulated the tyrosine kinase Src activity and tyrosine phosphorylation of several actin-binding proteins, including cortactin. This response was maintained over extended periods of time (for at least 60 min) (Durieu-Trautmann *et al.* 1994; Etienne *et al.* 1998; Etienne-Manneville *et al.* 2000). In order to assess whether PECAM-1 engagement might affect ICAM-1-induced cortactin phosphorylation, we performed the same sequential antibody cross-linking of ICAM-1 and PECAM-1 at the surface of RBE4 cells.

**Fig. 2:**
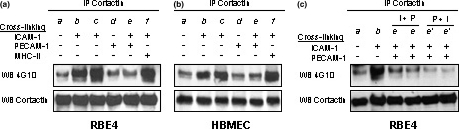
PECAM-1 cross-linking reverses ICAM-1-induced cortactin phosphorylation. Sequential cross-linking of ICAM-1 and/or PECAM-1 was performed on IFN-γ-treated RBE4 cells or HBMECs as described in Material and methods. Immunoprecipitated proteins were eluted with SDS-sample buffer, and resolved on 7.5% SDS-PAGE followed by immunoblotting with anti-phosphotyrosine (4 G10) or anti-cortactin antibodies. Panel (a) Cortactin immunoprecipitation using RBE4 cells. Panel (b) Cortactin immunoprecipitation using HBMECs. Lane a: basal condition; lanes b and c: ICAM-1 cross-linking (b, 5 μg/mL and c, 10 μg/mL of ICAM-1 antibodies); lane d: PECAM-1 cross-linking (10 μg/mL); lane e: sequential ICAM-1 and then PECAM-1 (10 μg/mL each) cross-linking; lane f: sequential ICAM-1 and then MHC class II cross-linking (10 μg/mL each). Panel (c) PECAM-1 cross-linking prevents ICAM-1-induced cortactin phosphorylation in RBE4 cells. Lane a: basal condition; lane b: ICAM-1 cross-linking (10 μg/mL of ICAM-1 antibodies); lane e (duplicates): ICAM-1 cross-linking followed by PECAM-1 cross-linking (I + P) (10 μg/mL antibodies); lane f (duplicates): PECAM-1 cross-linking followed by ICAM-1 cross-linking (P + I) (10 μg/mL antibodies).

As shown in Fig. 2, while ICAM-1 cross-linking strongly enhanced cortactin tyrosine phosphorylation in RBE4 cells (Fig. 2a, lanes b and c), PECAM-1 cross-linking, which had no effect alone (Fig. 2a, lane d), markedly reduced ICAM-1-induced cortactin phosphorylation (Fig. 2a, lane e). As a control, cross-linking of the IFN-γ -induced MHC class II proteins by an isotype-matched anti-MHC-II antibody (Fig. 2a, lane f) or of transferrin receptors (not shown) did not affect ICAM-1-induced cortactin phosphorylation. Moreover, similar results were observed using the non-brain endothelial cell line HBMEC (Fig. 2b). Partial inhibition of ICAM-1-induced cortactin phosphorylation was already observed after only 5 min of PECAM-1 cross-linking (not shown).

Furthermore, in order to assess whether PECAM-1 cross-linking could not only reduce ICAM-1-mediated response in RBE4 cells, but also prevent it, we then performed sequential cross-linking of PECAM-1 and ICAM-1 in this order. Figure 2c reveals that inhibition of ICAM-1-induced cortactin phosphorylation was even more drastic in this case (Fig. 2c, lanes P + I) than when PECAM-1 cross-linking followed ICAM-1 cross-linking as above (Fig. 2c, lanes I + P).

Altogether, these findings support the conclusion that PECAM-1 engagement on the surface of brain (and non-brain) endothelial cells drastically inhibits multiple ICAM-1 signaling pathways, including cortactin phosphorylation and actin rearrangements.

### The tyrosine phosphatase SHP-2 is at the crossroad between ICAM-1 and PECAM-1-coupled signaling pathways

**Fig. 3:**
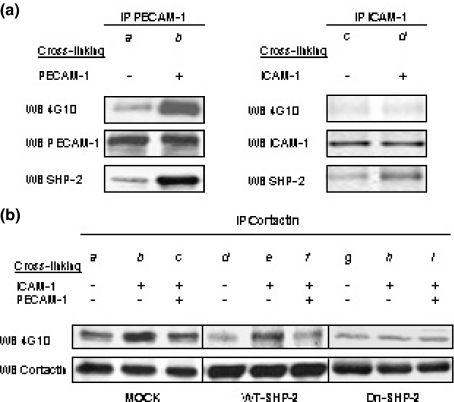
SHP-2 is recruited by cross-linked ICAM-1 and PECAM-1. Panel (a) Cross-linking of ICAM-1 or PECAM-1 was performed on IFN-γ-treated RBE4 cells as described in Material and methods. Immunoprecipitated proteins were eluted with SDS-sample buffer, and resolved on 7.5% SDS-PAGE followed by immunoblotting with anti-phosphotyrosine (4 G10) or anti-ICAM1 or PECAM-1 antibodies or with anti-SHP-2 antibody. Lane a: basal condition; lane b: PECAM-1 cross-linking; lane c: basal condition; lane d: ICAM-1 cross-linking. Panel (b) Sequential cross-linking of ICAM-1 and/or PECAM-1 was performed on IFN-γ-treated mock-transfected HBMECs or stably expressing WT-SHP-2 or Dn-SHP-2 as described in Material and methods. Immunoprecipitated proteins were eluted with SDS-sample buffer, and resolved on 7.5% SDS-PAGE followed by immunoblotting with anti-phosphotyrosine antibody (4 G10) or anti-cortactin antibody. Lanes a, d, g: basal condition; lanes b, e, h: ICAM-1 cross-linking; lanes c, f, i: sequential ICAM-1 and PECAM-1 cross-linking.

The tyrosine phosphatase SHP-2, is known to bind to activated PECAM-1. To gain insight into the molecular mechanisms mediating the observed inhibition of ICAM-1 signaling by PECAM-1 cross-linking, we then tested the hypothesis that SHP-2 might be involved in this process. Following PECAM-1 antibody cross-linking, we consistently observed a robust tyrosine phosphorylation of PECAM-1 and its interaction with SHP-2 as shown in Fig. 3a (lane b); the other known PECAM-1-interacting phosphatases SHP-1 and SHIP were not detected in the endothelial cells used in this study (data not shown).

**Fig. 4:**
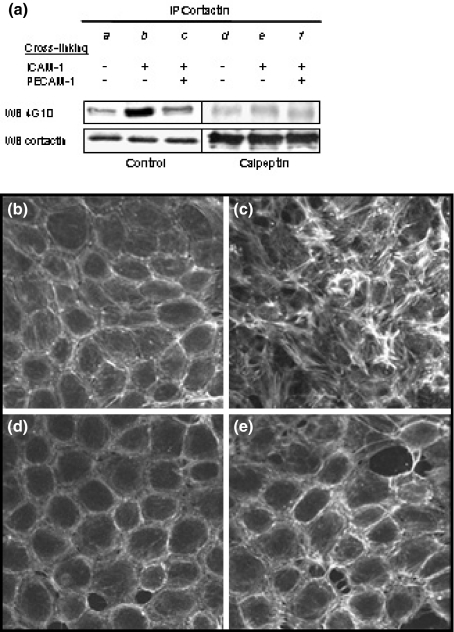
The SHP-2 inhibitor calpeptin mimicks PECAM-1-induced reversal of ICAM-1 signaling. Panel (a) Sequential cross-linking of ICAM-1 and/or PECAM-1 was performed on IFN-γ-treated RBE4 cells pre-incubated with or without the selective SHP-2 inhibitor calpeptin (150 μg/mL) as described in Material and methods. Following cortactin immunoprecipitation, immunoblotting with anti-phosphotyrosine antibodies (clone 4 G10) or anti-cortactin antibodies detected tyrosine-phosphorylated cortactin or total amount of cortactin, respectively. Lanes a, d: basal condition; lanes b, e: ICAM-1 cross-linking; lanes c, f: sequential ICAM-1 and PECAM-1 cross-linking. Panels (b–e) IFN-γ-treated RBE4 cells were treated or not with calpeptin (150 μg/mL for 30 min) prior to ICAM-1 cross-linking. Cells were fixed and actin was stained by using 0.1 μg/mL of tetramethylrhodamine isothiocyanate-labeled phalloidin. Panel (b) Basal condition. Panel (c) ICAM-1 cross-linking. Panel (d) Basal condition after calpeptin treatment. Panel (e) ICAM-1 cross-linking after calpeptin treatment.

Moreover, focusing on ICAM-1 signaling pathway, we observed a slight but consistent increase in the amount of SHP-2 in ICAM-1 immunoprecipitates following antibody cross-linking, although we failed to detect any ICAM-1 phosphorylation (Fig. 3a, lane d): this result suggested a non-conventional or indirect interaction between ICAM-1 and SHP-2. Similar results were observed using HBMECs (not shown). To further assess a functional role for SHP-2 in ICAM-1-coupled signaling pathways, we established endothelial cell lines stably expressing a dominant negative form of SHP-2 (Dn-SHP-2) or the wild type enzyme (WT-SHP-2) as a control; for technical reasons, HBMECs were used in this approach. Following antibody cross-linking of ICAM-1, we observed that expression of Dn-SHP-2 abolished the ICAM-1 induced tyrosine phosphorylation of cortactin (Fig. 3b, lane h), pointing to SHP-2 as a positive regulator of the Src-cortactin pathway in endothelial cells. By contrast, over-expression of the wild type enzyme had no effect (Fig. 3b, lane e), suggesting that endogenous SHP-2 activity was sufficient for a maximal stimulation of cortactin phosphorylation by ICAM-1 cross-linking. Immunoblotting with anti SHP-2 antibody (not shown) was used for control of sample loading. Interestingly, calpeptin (Fig. 4a, lane e), a known calpain inhibitor which was previously identified and characterized as a selective inhibitor of SHP-2 activity (Schoenwaelder *et al.* 2000; Gopalakrishnan *et al.* 2003) drastically affected the formation of actin stress fibers in response to antibody cross-linking of ICAM-1 in RBE4 cells (Fig. 4e) and in HBMECs (not shown).

**Fig. 5:**
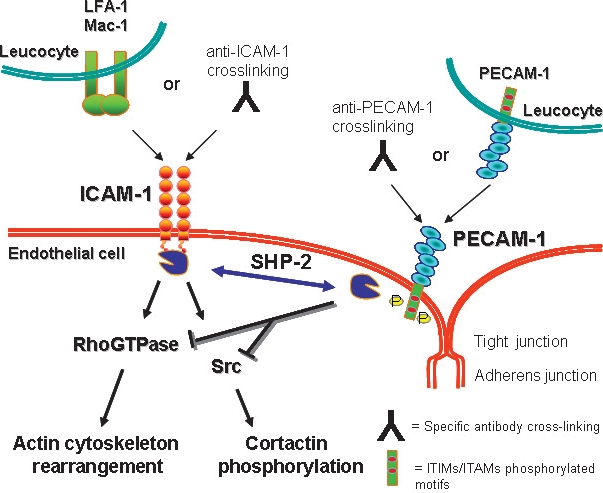
Cross-talk between ICAM-1 and PECAM-1-coupled signaling pathways. SHP-2 is positively involved in ICAM-1 signaling, upstream of the tyrosine phosphorylation of cortactin by Src and of RhoA-mediated actin cytoskeleton rearrangements. In this study, we show that PECAM-1 engagement, which induces a strong interaction with SHP-2, drastically inhibits these two distinct ICAM-1 activated pathways. Together, we propose that SHP-2 might be a key player at the crossroads between ICAM-1 and PECAM-1 signalings in endothelial cells.

In conclusion, the main findings of the current study are summarized in Fig. 5: they demonstrate that SHP-2 is positively involved in ICAM-1 signaling, upstream of the tyrosine phosphorylation of cortactin by Src and of RhoA-mediated actin cytoskeleton rearrangements. More importantly, our results show that PECAM-1 engagement, which induces a strong interaction with SHP-2, drastically inhibits these two distinct ICAM-1 activated pathways. Together, these results suggest that SHP-2 might be a key player at the crossroads between ICAM-1 and PECAM-1 signalings in endothelial cells.

## Discussion

We show in the present study that PECAM-1 engagement by antibody cross-linking totally blocked ICAM-1-induced activation of RhoA and subsequent changes in actin cytoskeleton, as well as tyrosine phosphorylation of cortactin in rat brain endothelial cells. Moreover, these findings suggest that the tyrosine phosphatase SHP-2, as an upstream mediator of both PECAM-1 and ICAM-1 signaling pathways, might be a key player in the cross-talk identified here between these pathways.

Accumulating evidence indicates that, beyond its ability to contribute to the formation of “docking” sites for circulating leucocytes (Barreiro *et al.* 2002; Carman *et al.* 2003), ICAM-1 behaves as a signal transducer, activating multiple signaling pathways in endothelial cells. ICAM-1 signaling in endothelial cells thus appears to participate in the trans-endothelial migration of leucocytes to inflammation sites. We previously identified multiple signaling pathways activated in rat brain endothelial cell lines, following ICAM-1 cross-linking or leukocyte adhesion. In particular, we demonstrated activation of the tyrosine kinase Src and tyrosine phosphorylation of several cytoskeleton-associated proteins, including cortactin (Etienne *et al.* 1998; Adamson *et al.* 1999; Etienne-Manneville *et al.* 2000). In spite of the short C-terminal domain of ICAM-1, which comprises 27 amino acids in rat and 28 amino acids in human, biochemical studies have demonstrated that ICAM-1 is capable of binding a number of intracellular proteins in endothelial cells: the actin-binding protein α-actinin, the microtubule-associated β-tubulin, the enzyme glyceraldehyde-3-phosphate dehydrogenase and ezrin, a linker between integral membrane proteins and the actin-cytoskeleton (Federici *et al.* 1996; Celli *et al.* 2006). In addition, it has been suggested that SHP-2 might associate with tyrosine phosphorylated ICAM-1, even though ICAM-1 does not contain a *bona fide* SHP-2 binding site, such as a consensus immunoreceptor tyrosine-based inhibitory motif (Pluskota *et al.* 2000; Wang *et al.* 2003). In the present study, the moderate amount of SHP-2 bound to ICAM-1 (compared with SHP-2 bound to PECAM-1) following antibody cross-linking may be related to a very low level of ICAM-1 tyrosine phosphorylation (not detected here or in a previous studies (Greenwood *et al.* 2003)) or may reflect interaction with a putative non-conventional motif in the ICAM-1 cytoplasmic domain. Alternatively, interaction between ICAM-1 and SHP-2 might be indirect, via an unidentified protein linker.

SHP-2 is known as an upstream mediator of multiple signaling pathways, but contrasting data in various cell systems present it either as a negative or a positive regulator of RhoA and other pathways (Pluskota *et al.* 2000; Lacalle *et al.* 2002; Wang *et al.* 2003; Kontaridis *et al.* 2004). Here we showed that a dominant negative form of SHP-2 (Dn-SHP-2) or calpeptin inhibition of SHP-2 enzymatic activity largely inhibited the ICAM-1-mediated tyrosine phosphorylation of cortactin and actin stress fiber formation. These results, summarized in Fig. 5, clearly establish that SHP-2 is positively involved in ICAM-1 signaling in endothelial cells, upstream of the Src-mediated tyrosine phosphorylation of cortactin as well as RhoA-mediated actin cytoskeleton rearrangements.

PECAM-1 has been convincingly shown to transduce inhibitory signals in a variety of cell types, including endothelial cells, through SHP-2 binding to a tyrosine-phosphorylated immunoreceptor tyrosine-based inhibitory motif in its cytoplasmic domain (Masuda *et al.* 1997; Henshall *et al.* 2001; Patil *et al.* 2001; Cicmil *et al.* 2002; Gratzinger *et al.* 2003; Jackson 2003; Newman and Newman 2003). The results of the present study showed that PECAM-1 engagement concomitantly induced SHP-2 binding to PECAM-1 and blocked the SHP-2-dependent phosphorylation of cortactin and rearrangements of the actin cytoskeleton evoked by ICAM-1. This is to our knowledge the first evidence that PECAM-1 engagement can negatively regulate the signaling pathways coupled to another leukocyte adhesion molecule in endothelial cells. This observation strongly suggests that leukocyte adhesion and transmigration trigger in endothelial cells a complex set of interconnected positive and negative signalings which cooperate for controlling the dynamics of endothelial response. Although our study cannot firmly establish a direct role for SHP-2 in PECAM-1-coupled inhibition of ICAM-1 signaling, it is tempting to speculate that activated PECAM-1, by efficiently recruiting SHP-2, may sequester it away from ICAM-1 and inhibit RhoA and Src activation. This hypothesis is in line with the interpretation proposed for PECAM-1 inhibition of growth factor or antigen receptor signaling pathways (Newman *et al.* 2001; Newman and Newman 2003). Alternatively, other proteins involved in the maintenance of endothelial junction integrity such as β- or γ-catenin, PLC-γ, which are also known to interact with the cytosolic domain of PECAM-1, might be involved in the regulatory loop identified here between ICAM-1 and PECAM-1 (Ilan and Madri 2003). However, we did not observe in the present study any detectable association between PECAM-1 and β- or γ-catenin (not shown).

We and others provided compelling evidence that ICAM-1-induced signaling and subsequent actin cytoskeleton remodeling was directly involved in the process of trans-endothelial migration of activated leukocytes (Durieu-Trautmann *et al.* 1994; Greenwood *et al.* 2002; Carman and Springer 2004; Shaw *et al.* 2004; Yang *et al.* 2005; Millan *et al.* 2006). Pharmacological inhibition of ICAM-1 coupled signalling pathways, using Rho kinase inhibitors or calcium chelators, largely prevented the migration of activated lymphocytes across RBE4 cells (Adamson *et al.* 1999; Etienne-Manneville *et al.* 2000). Moreover, we demonstrated that inhibition of Rho GTPases with protein prenyltransferase inhibitors prevented leukocyte recruitment to the CNS and attenuated clinical signs of disease in Experimental Autoimmune Encephalomyelitis (Walters *et al.* 2002). Complementary experiments demonstrated that the ICAM-1-induced Src-cortactin pathway is essential for trans-endothelial migration of leukocytes (Yang *et al.* 2005, 2006).

Moreover, recent studies have demonstrated that leukocyte adhesion promotes the remodeling of the apical endothelial plasma membrane into projections that surround adherent leukocytes (Carman and Springer 2004; Shaw *et al.* 2004; Barreiro *et al.* 2005; Doulet *et al.* 2006). These structures, referred to as endothelial docking structures or transmigratory cups, are essential to promote firm adhesion and extravasation of leukocytes through paracellular as well as transcellular routes. Formation of these docking structures results from the dynamic redistribution of several adhesion molecules including ICAM-1 and actin polymerization. The current study strongly suggests that PECAM-1 engagement during paracellular extravasation of leukocytes, which has been well-documented to contribute to leukocyte diapedesis, may also regulate this process by limiting the ICAM-1 induced actin cytoskeleton rearrangements, downstream of the formation of docking structures. Future studies will aim at evaluating the functional relevance of these findings, by evaluating the role of the PECAM-1 cytoplasmic domain and associated signaling in regulation of leukocyte transmigration across brain endothelial cells. In addition, because PECAM-1 is not present in endothelial docking structures, it will be necessary to identify the cellular compartments where the signalling cross-talk reported here may take place. It can be hypothesized that caveolae or caveolin-rich microdomains may facilitate the functional interaction between ICAM-1- and PECAM-1 signalings in as much as PECAM-1 is present in these structures whereas ICAM-1 was recently shown to be recruited in similar structures following antibody cross-linking (Millan *et al.* 2006; Viegas *et al.* 2006). Further experiments will be required to substantiate this hypothesis.

## Abbreviations used

HBMEC: human bone marrow endothelial cell line
ICAM-1: intercellular adhesion molecule-1
MHC: major histocompatibility complex
PECAM-1: platelet/endothelial cell-adhesion molecule-1
RBE4: rat brain endothelial cell line 4
